# Identification and Analysis of Circular RNAs in Mammary Gland from Yaks Between Lactation and Dry Period

**DOI:** 10.3390/ani15010089

**Published:** 2025-01-03

**Authors:** Yilin Shi, Xiaoyun Wu, Guangyao Meng, Xiaoming Ma, Yongfu La, Pengjia Bao, Min Chu, Ping Yan

**Affiliations:** 1 Key Laboratory of Yak Breeding of Gansu Province, Lanzhou Institute of Husbandry and Pharmaceutical Sciences, Chinese Academy of Agricultural Sciences, Lanzhou 730050, China; shi_yilin37@163.com (Y.S.); wuxiaoyun@caas.cn (X.W.); mgy19971104@163.com (G.M.); maxiaoming@caas.cn (X.M.); layongfu@caas.cn (Y.L.); baopengjia@caas.cn (P.B.); 2 Key Laboratory of Animal Genetics and Breeding on Tibetan Plateau, Ministry of Agriculture and Rural Affairs, Lanzhou 730050, China; 3 Institute of Western Agriculture, Chinese Academy of Agricultural Sciences, Changji 931100, China

**Keywords:** yak, circRNA, ceRNA, mammary gland, transcriptome

## Abstract

Lactation is a complex physiological process regulated by numerous genes and factors. Circular RNA (circRNA), a non-coding RNA, acts as a molecular sponge that sequesters microRNAs (miRNAs) to regulate target gene expression. Although circRNA has been linked to mammary gland lactation, its specific role in yaks remains underexplored. This study employed circular RNA sequencing (circRNA-seq) to examine the differential expression of circRNAs in yak mammary tissues during lactation and the dry period. Additionally, an enrichment analysis of the differentially expressed circRNAs (DECs) was performed. A competing endogenous RNA (ceRNA) network was then constructed to explore the potential of their roles in lactation and mammary gland development. We detected 18,905 circRNAs in yak mammary tissue, among which 302 showed differential expression. The host genes of these DECs were enriched in functions and pathways associated with yak milk synthesis and composition. Through the construction of a ceRNA network and the enrichment analysis of associated mRNAs, this study identified ceRNAs potentially involved in regulating lactation and mammary gland development. In conclusion, circRNAs in yak mammary tissues were identified and analyzed across lactation and dry periods, establishing a ceRNA network related to lactation regulation. These findings provide novel insights into the regulatory mechanisms governing lactation in yaks (*Bos grunniens*).

## Simple Summary

Circular RNA (circRNA), a non-coding RNA that regulates gene expression, plays significant biological roles. Understanding the role of yak lactation and mammary gland development is crucial. This study conducted Circular RNA sequencing (circRNA-Seq) analysis of yak mammary tissues during the lactation (LP) and dry period (DP) stages. A total of 18,905 circRNAs were identified in yak mammary tissues, and the differentially expressed circRNAs (DECs) may contribute to milk synthesis and composition regulation. This study provides a valuable resource for enhancing yak circRNA databases, marking its particular significance.

## 1. Introduction

The yak, primarily found in the harsh alpine steppe of the Qinghai-Tibet Plateau, is a crucial livestock species in this region, providing essential resources such as meat, milk, and fiber. Yak milk, often called “natural concentrated milk”, is a crucial source of essential nutrients for individuals in high-altitude, low-oxygen regions. Compared to cow’s milk, yak milk contains higher levels of protein, fatty acids, antioxidant vitamins, and other beneficial components [1,2], classifying it as high-quality milk. However, the milk yield of yak is relatively low, with a single lactation producing only 150–500 kg [3], which falls short of meeting industry demands. Consequently, increasing yak milk yield is an important goal for yak breeding.

The mammary gland is a unique organ in mammals, playing a crucial role in the reproductive process of mammals. Unlike other organs, most mammary gland tissue development occurs postnatally [4,5]. Pregnancy facilitates the final stages of mammary gland development, enabling the organ’s functional capacity to secrete milk by the end of gestation. During lactation, the mammary gland undergoes the cycles of cell proliferation, differentiation, dedifferentiation, and apoptosis, all regulated by various hormones and regulatory genes [6,7]. These processes are complex and involve extensive gene transcription regulation. CircRNA is a unique endogenous non-coding RNA that lacks 5′ and 3′ ends, forming a closed circular structure stabilized by covalent bonds [8,9,10]. The unique structure and distinct localization of circRNAs endow them with specific biological functions. Most circRNAs act as miRNA sponges, inhibiting miRNAs’ regulatory effects on target genes and thereby influencing target gene expression levels [11,12]. CircRNAs have been implicated in the regulation of various physiological processes, including mammary gland development and lactation. For example, circEZH2 influences cell proliferation, apoptosis, and lipid metabolism in bovine mammary epithelial cells [13]. Circ-016910 affects anti-apoptotic and proliferative functions in goat mammary epithelial cells and regulates β-casein and triglyceride secretion via miR-574-5p [14]. In bovine mammary epithelial cells, circRNA-11228 binds to miR-103, inhibiting expression of the target gene INSIG and regulating milk fat production [15]. CircMYL1, as a molecular sponge for miR-2400, can eliminate its inhibitory effect on myoblast differentiation [16]. CircTTN activates the IGF2/PI3K/AKT signaling pathway by competitively binding to miR-432, thereby promoting the proliferation and differentiation of bovine primary myoblasts [17].

Given the role of circRNA in mammary gland development and lactation in mammals, we selected the yak as a research model and used high-throughput circRNA-Seq to study circRNA in mammary tissue during lactation and dry periods. The aim of this study was to better understand the role that mammary circrnas play during yak lactation and how non-coding RNAs act in combination during this period. This study offers new insights into the regulatory mechanisms of lactation and mammary gland development in yaks.

## 2. Materials and Methods

### 2.1. Ethics Statement

All experiments were conducted in accordance with the Guidelines for the Care and Use of Laboratory Animals (Approval No. 2006-398) formulated by the Ministry of Science and Technology, PRC. All experimental protocols and procedures were approved by the Animal Management Ethics Committee of the Lanzhou Institute of Animal Science and Veterinary Medicine, Chinese Academy of Agricultural Sciences (Permit No. SYXK-2014-0002).

### 2.2. Animal and Sample Collection

We selected five healthy female Ashdan yaks from Datong Cattle Farm, Qinghai Province. All animals grazed freely on natural pastures and were second-born yaks. Due to sampling limitations, we selected only two yaks at peak lactation (LP; 120 days postpartum, non-pregnant) and three yaks in the dry period (DP; calved the previous year, non-pregnant) for sampling. Immediately after slaughter, the right posterior mammary gland of each yak was incised, and alveolar tissue from the upper middle third of the gland was extracted and stored in liquid nitrogen.

### 2.3. RNA Extraction and Quality Control

Total RNA was extracted from mammary gland tissue using TRIzol reagent (Invitrogen, Carlsbad, CA, USA), following the manufacturer’s protocol. The quality and purity of total RNA were evaluated using a nanophotometer spectrophotometer (IMPLEN, Munich, Germany), and integrity was confirmed with a Bioanalyzer 2100 system (Agilent Technologies, Palo Alto, CA, USA) and the RNA Nano 6000 assay kit (Agilent Technologies, Santa Clara, CA, USA). Each sample showed a 260/280 ratio of approximately 2.0, and the RNA integrity number (RIN) exceeded 8.0.

### 2.4. Library Construction and Sequencing

After rRNA removal with the Ribo-Zero rRNA Removal kit (Epicentre Biotechnologies, Madison, WI, USA), linear RNA digestion was performed on the recovered RNA using RNase R (Epicentre Biotechnologies, Madison, WI, USA) to prepare cDNA. The synthesized cDNA underwent purification and end-repair, followed by 3′-end adenylation and adaptor ligation with the NEBNext Ultra RNA Library Prep Kit for Illumina (New England Biolabs Inc., Ipswich, MA, USA). After PCR amplification and purification, library quality was assessed using the TapeStation System (Agilent Technologies, Palo Alto, CA, USA). Sequencing was conducted on an Illumina HiSeq 2000 platform (Illumina, Inc., San Diego, CA, USA) with the 150 bp paired-end model.

### 2.5. Identification and Annotation of circRNAs

The identification of circRNAs followed as described by Zhang et al. [18], with slight modifications. First, the quality of the raw data was assessed using C++ (v20) and R (v4.3.2). Based on quality assessment results, Cutadapt (v1.16) [19] was used to remove sequencing adapters, and FASTX-Toolkit (v0.0.13) [20] was used for data quality control. C++ and R were then used again for quality assessment, followed by rRNA sequence removal with TopHat2 (v2.0.9) [21], and the test was repeated to obtain clean data. The rRNA-depleted reads were then mapped to the yak reference genome (BosGru_v2.0) using TopHat2 (v2.0.9) without fusion gene alignment. TopHat-Fusion (v2.0) [22] was used to align unmapped reads with the reference genome for fusion genes, and fusion alignment results were used to select candidate back-spliced junction reads at splice sites matching the exon-loop RNA structure. Candidate reads not matching splice site sequence characteristics were filtered out, and the circRNAs were then identified. For identified circRNAs, functional elements (e.g., exons, introns, splice sites, 5′ UTRs, 3′ UTRs, etc.) were annotated using Python.

### 2.6. Differentially Expressed circRNAs and miRNAs

The RPM value of each circRNA and miRNA was calculated to quantify circRNAs and miRNAs using HTseq Software (v2.0.3) with the following formula:

$$RPM=\frac{number\;of\;reads\;mapping\;to\;circRNAs}{number\;of\;reads\;in\;clean\;data}\times 10^{6}$$

In this formula, the number of reads mapping to circRNAs and miRNAs includes both TopHat2 (v2.0.9) and TopHat-Fusion (v2.0) mapped reads. Differential expression analysis was performed with the DEGSeq, and differential circRNAs with |log_2_ (Fold Change)| ≥ 1, q-values < 0.001 were further analyzed.

### 2.7. Target miRNAs and Genes Prediction and ceRNA Network Analysis

In this study, miRanda Software (v3.3a) [23] was used to predict potential miRNA binding sites for all circRNAs. RNAhybrid (v2.2) [24], PITA [25], and miRanda (v3.3a) [23] were used concurrently to predict putative miRNA-mRNA interactions. Our miRNA library data were deposited in the NCBI Sequence Read Archive (SRA) (Accession code: PRJNA1186264; unpublished data) using the same samples. Differential mRNA data were sourced from our group’s published papers [26]. Based on the competitive endogenous RNA (ceRNA) hypothesis, circRNA–miRNA–mRNA pairs were developed according to the following steps: (1) All DEcircRNAs, DEmRNAs, and differentially expressed miRNAs (DEmiRNAs) were retained; (2) The circRNA–miRNA–mRNA network contains two regulatory patterns: one in which down-regulated miRNAs are paired with up-regulated circRNAs and mRNAs, and the other where up-regulated miRNAs are paired with down-regulated circRNAs and mRNAs.

### 2.8. GO and KEGG Enrichment Analysis

Kyoto Encyclopedia of Genes and Genomes (KEGG) enrichment analysis of host genes of differentially expressed circRNAs and target genes within ceRNA networks was conducted using the KOBAS Web Tool [27] with a hypergeometric test, while Gene Ontology (GO) analysis was conducted using the G:Profile Web Tool [28], and *p*-value correction was performed using Benjamini–Hochberg method. Pathways with a gene count ≥ 2 and a *p*-value < 0.05 were considered significant.

### 2.9. Verification of Sequencing Data Using qRT-PCR

The relative expression of randomly selected circRNAs was assessed to verify the accuracy of the RNA-seq results using quantitative real-time PCR (qRT-PCR). Following the manufacturer’s protocol, RNA was reverse-transcribed into cDNA using the Transcriptor First Strand cDNA Synthesis Kit (Roche Diagnostics, Mannheim, Germany). Hydroxymethylbilane synthase (HMBS) and tyrosine 3-monooxygenase/tryptophan 5-monooxygenase activation protein, zeta polypeptide (YWHAZ) were used as reference genes. The 20 μL PCR reaction system contained 1 μL DNA, 1 μL forward primer, 1 μL reverse primer, 10 μL SYBR TB Green mix (TaKaRa, Dalian, China), and 7 μL ddH_2_O. All qRT-PCR reactions were conducted on the LightCycler 96 Instrument (Roche, Basel, Switzerland), with three technical replicates for each sample. Four circRNAs were randomly selected for validation, and their primers were designed using the Primer3 Plus Software (v3.3.0) [29]. Relative RNA expression levels were calculated based on the cycle threshold (Ct) using the 2^−ΔΔCt^ method [30].

## 3. Results

### 3.1. RNA-Seq Data Analysis

Five cDNA libraries were constructed for circRNA-seq to detect circRNA in yak mammary glands. Statistical results showed that a total of 802,296,424 raw paired-end reads were obtained from the five samples. After removing low-quality reads and adaptor sequences, 784,047,750 clean reads were obtained (DP: 491,853,434; LP: 292,194,316). Moreover, 83.79–86.48% of the clean reads were mapped to the yak reference genome using Tophat2 (v2.0.9) software. The GC content ranged from 50.51% to 53.23%, and the Q30 content from 96.35% to 96.53% (Table 1). Correlation analysis showed a strong correlation between individuals (Figure S1), demonstrating that the expression patterns of circRNA across the five yak mammary gland samples were strongly consistent with one another.

### 3.2. Identification of circRNAs

**Figure 1:**
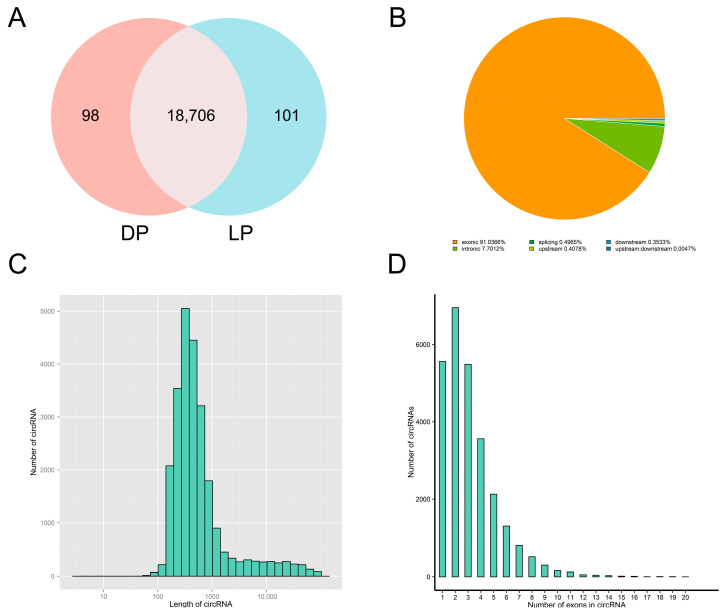
Identification and characterization of circRNAs. (**A**) Specific and shared circRNAs between DP and LP groups; (**B**) Structural types of circRNAs; (**C**) Length distribution of circRNAs; (**D**) Exon counts within circRNAs.

After rigorous screening and filtering, a total of 18,905 circRNAs were detected in the mammary tissue of yaks, with 98 circRNAs specific to the DP group and 101 specific to the LP group (Figure 1A). Based on genomic location, 91.04% of the circRNAs were classified as exonic circRNAs and 7.70% as intronic circRNAs (Figure 1B). The length of most circRNA was about 300 to 600 nt (Figure 1C). The number of exons within circRNAs ranged from 1 to 20, with the majority clustered between 1 and 5 exons (Figure 1D).

### 3.3. Differentially Expressed circRNAs Between DP and LP Groups

**Figure 2:**
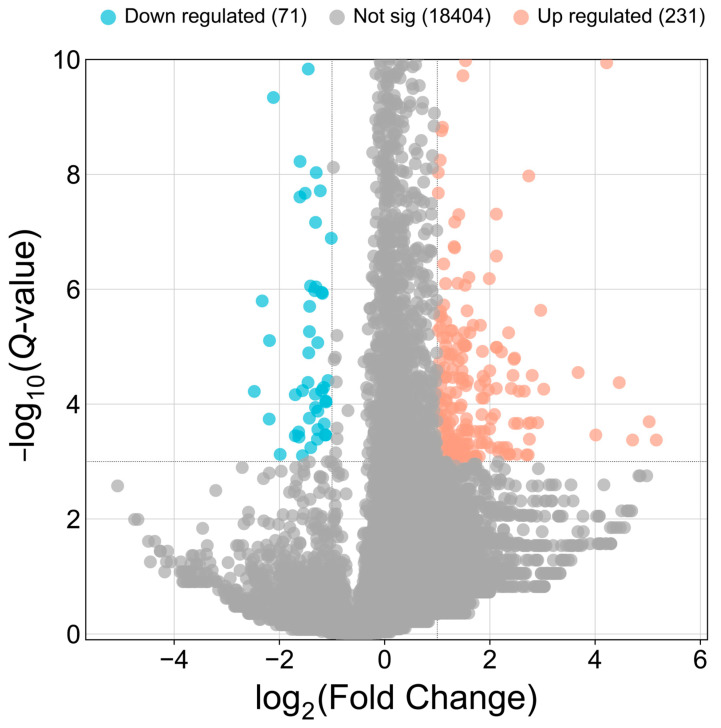
Differentially expressed circRNAs in yak mammary tissues during lactation and dry periods. Note: Orange and blue dots indicate up-regulated and down-regulated circRNAs, respectively.

Through quantitative circRNA analysis, we identified a total of 302 differentially expressed circRNAs (DECs). Of these, 231 circRNAs showed significant up-regulation, while 71 were significantly down-regulated in which group. This differential expression pattern suggests that these circRNAs may play regulatory roles in key biological processes across varying physiological conditions (Figure 2).

### 3.4. Functional Enrichment Analysis of Host Gene

**Figure 3:**
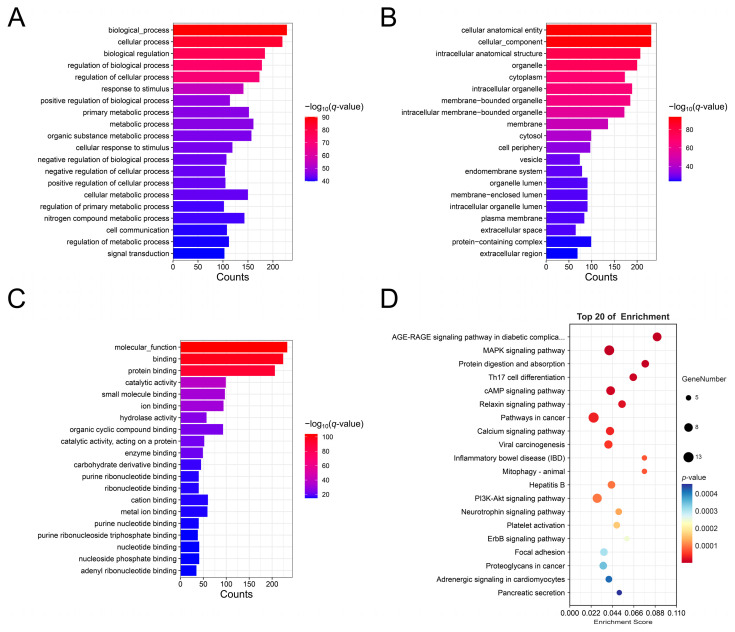
GO and KEGG enrichment analyses of host genes for DECs. (**A**) Top 20 GO pathways for biological processes; (**B**) Top 20 GO pathways for cellular components; (**C**) Top 20 GO pathways for molecular functions; (**D**) Top 20 KEGG pathways.

A total of 277 host genes correspond to 302 DE circRNAs. GO enrichment analysis showed that the host genes were enriched in a total of 2142 terms: 1615 under biological processes (BP), 257 under cellular components (CC), and 270 under molecular functions (MF) (q-value < 0.05). The results indicate that the top 20 BP terms enriched for host genes of DECs include biological_process, cellular process, biological regulation, etc. (Figure 3A). The top 20 CC items include a cellular, anatomical entity, cellular_component, intracellular anatomical structure, etc. (Figure 3B). The top 20 MF items include molecular_function, binding, protein binding, etc. (Figure 3C). Additionally, we annotated entries related to mammary development, including mammary gland epithelium development, mammary gland development, and mammary gland duct morphogenesis (Table S1). KEGG enrichment analysis indicated that host genes of DECs were significantly enriched in 117 pathways (Table S2). The top 20 KEGG pathways are shown in Figure 3D, including the AGE-RAGE signaling pathway in diabetic complications, MAPK signaling pathway, protein digestion and absorption, etc.

### 3.5. Construction of ceRNA Network

**Figure 4:**
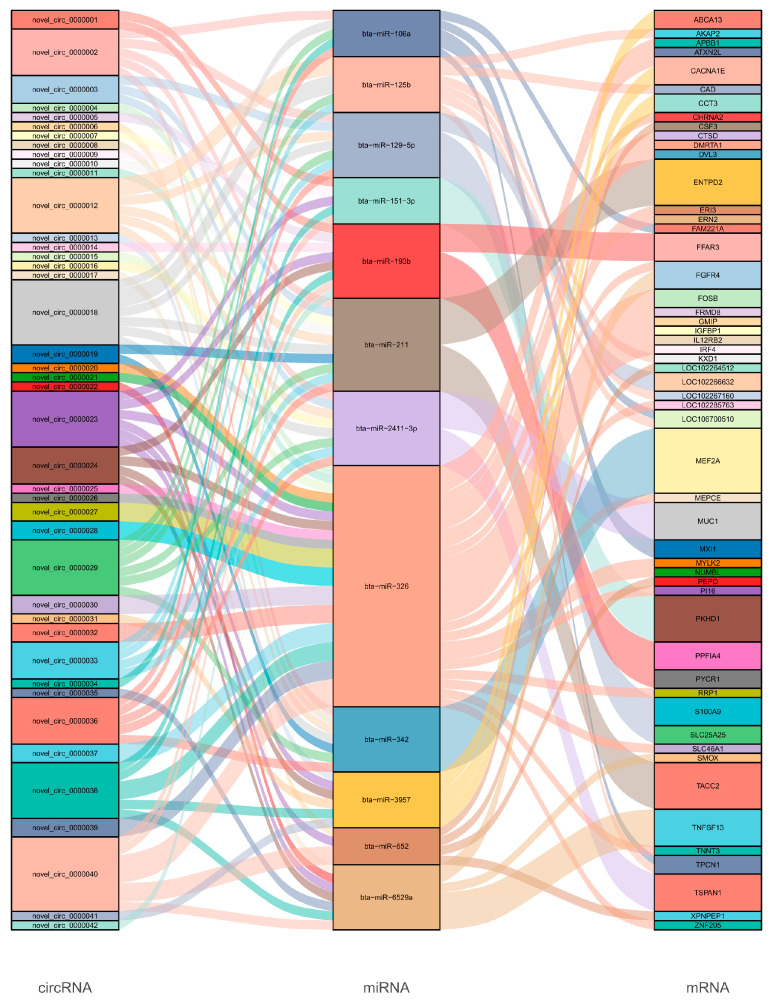
Sankey diagram representing the ceRNA network in mammary tissues of the yak.

**Figure 5:**
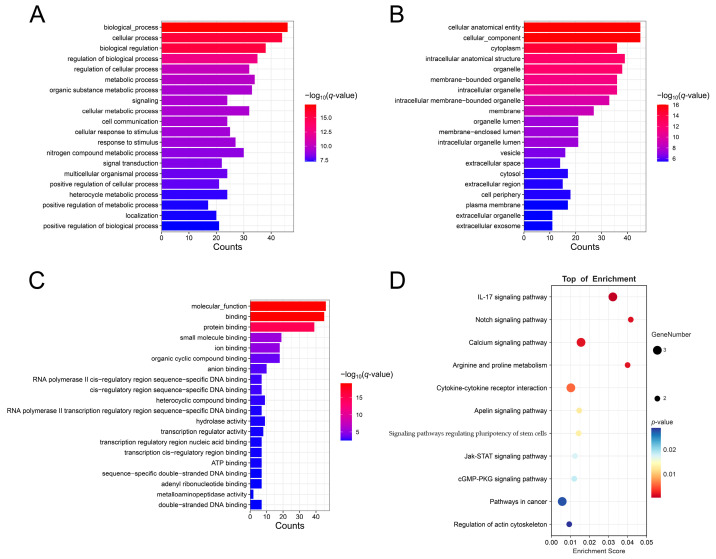
GO and KEGG enrichment analyses of target genes of DEmiRNAs in the ceRNA network. (**A**) Top 20 GO pathways for biological processes; (**B**) Top 20 GO pathways for cellular components; (**C**) Top 20 GO pathways for molecular functions; (**D**) Most enriched KEGG pathways.

According to the ceRNA hypothesis, circRNAs can regulate mRNA expression by acting as miRNA sponges through their miRNA binding sites. We combined our miRNA data (unpublished) to construct the circRNA–miRNA–mRNA regulatory network. The ceRNA network contained 87 circRNA–miRNA pairs and 58 miRNA–mRNA pairs, including 42 DEcircRNAs, 12 DEmiRNAs, and 53 DEmRNAs (Figure 4). The target genes of DEmiRNAs in the ceRNA network were significantly enriched in 584 GO terms (q-value < 0.05, Table S3). The top 20 BP-enriched terms include biological_process, cellular process, biological regulation, etc. (Figure 5A). The top 20 CC-enriched terms include a cellular, anatomical entity, cellular_component, cytoplasm, etc. (Figure 5B). The top 20 MF-enriched terms include molecular_function, binding, protein binding, etc. (Figure 5C). A total of 11 KEGG pathways were enriched for target genes in the ceRNA network, including the IL-17 signaling pathway, Notch signaling pathway, calcium signaling pathway, etc. (Figure 5D).

### 3.6. Real-Time Quantitative PCR Validation of Sequencing Data

**Figure 6:**
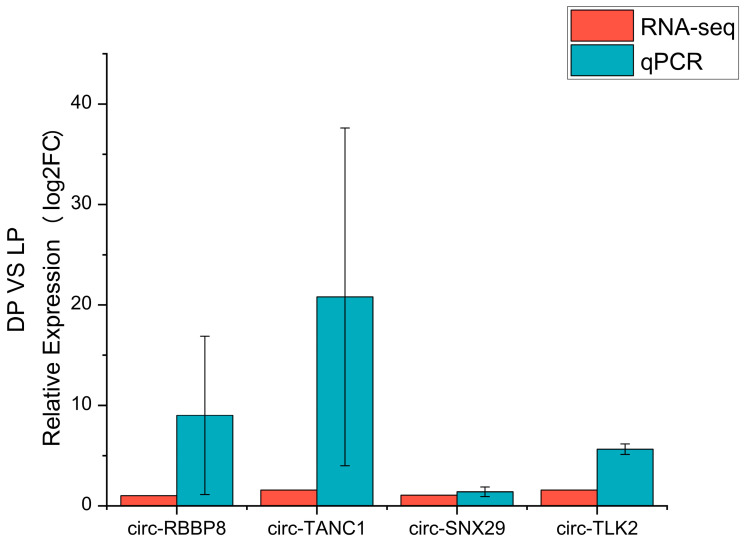
Expression patterns of circRNAs: Validation and comparison of log_2_(Fold Change) between qRT-PCR and circRNA-Seq.

Four DECs were randomly selected for validation by qRT-PCR. These DECs were derived from four host genes: retinoblastoma binding protein 8 (*RBBP8*), tetratricopeptide repeat, ankyrin repeat, and coiled-coil containing 1 (*TANC1*), sorting nexin-29 (*SNX29*) and tousled-like kinase 2 (*TLK2*). Details of the primers are presented in Table S4. The results showed that all four DECs exhibited expression patterns consistent with the RNA-seq data, supporting the reliability of circRNA-seq results in this study (Figure 6).

## 4. Discussion

To explore the physiological roles of circRNAs in yak mammary glands, we analyzed circRNAs in the mammary tissue of two yaks at peak lactation and three yaks in the dry period using transcriptome sequencing. Over 83.79% of the clean RNA-seq data were mapped to the yak reference genome, consistent with previous RNA-seq studies on yak blastocysts [31].

In this study, 18,905 circRNAs were detected in the mammary tissue of yaks in the lactation and dry period. Ma et al. [32] identified a total of 5534 circRNAs in the *longissimus dorsi* muscle. La et al. [33] identified a total of 16,185 circRNAs in yak testis. The variation in circRNA numbers may be due to tissue-specific expression. In this study, the circRNAs detected in the mammary tissue of yaks mainly originated from exons, which is consistent with results from the mammary tissue of Holstein cows [34], sheep [35], and goats [36,37]. Most circRNAs identified in yak mammary tissue were less than 1 kb in length, with a predominant distribution in the 300–600 bp range. This is consistent with the length distribution of circRNAs in Holstein cow mammary tissue [34] and goat mammary tissue [37]. Our results show consistency with circRNA data identified from other species, showing high reliability.

GO and KEGG enrichment analyses characterize the primary functions of genes. GO annotation revealed that the host genes of DE circRNAs were primarily enriched in metabolic processes, including metabolic process, organic substance metabolic process, and cellular metabolic process. KEGG enrichment analysis identified that the host genes were annotated to pathways, including the MAPK signaling pathway, protein digestion and absorption, cAMP signaling pathway, and calcium signaling pathway, all of which are closely related to lactation. The MAPK signaling pathway is involved in the regulation of many important physiological processes, including milk synthesis [14]. Changes in intracellular calcium concentrations can influence the production or degradation of cAMP, and the cAMP signaling pathway plays a critical role in regulating the expression of β-casein in human mammary epithelial cells [38,39].

CircRNA is a non-coding RNA that acts as a sponge, sequestering endogenous miRNAs and thereby protecting downstream mRNAs from miRNA-mediated repression. A study on circ09863 in breast epithelial cells showed that overexpression of circ09863 significantly countered the inhibitory effect of miR-27a-3p on its target gene FASN, promoting FASN expression to regulate TAG synthesis and fatty acid composition [40]. Similarly, circ01592 binds miR-218, enhancing the expression of its target gene ELOVL5 and promoting TAG synthesis and fatty acid composition [41]. Similar to host genes, GO enrichment analysis revealed that the target genes in the ceRNA network were mainly involved in metabolic processes, including organic substances and cellular metabolic processes. KEGG enrichment analysis also annotated the target genes to lactation-related signaling pathways. MYLK2, TPCN1, and CACNA1E were mapped to the calcium signaling pathway, while PYCR1 and SMOX were mapped to the arginine and proline metabolism pathway. Arginine has been shown to support the proliferation of bovine mammary epithelial cells and to regulate the transcription of casein genes and pathway-related genes [42,43]. Arginine catabolism to proline can lead to polyamine synthesis in placental tissue during pregnancy [44]. In the ceRNA network examined in this study, MUC1 is associated with mastitis resistance and milk fat synthesis [45]. Target genes of bta-miR-193b included FFAR3 and PYCR1, and bta-miR-193b was significantly down-regulated in the LP group. FFAR3, previously known as GPR41, is a G-protein-coupled cell surface receptor for short-chain fatty acids (SCFAs) that primarily mediates SCFA signal transduction in bovine mammary epithelial cells [46]. Additionally, the FFAR3 gene is involved in lipid metabolism, according to CNVR annotation [47]. PYCR1 promotes intracellular amino acid metabolism during lactation and is associated with L-proline synthesis [48]. Bta-miR-326, which targets and regulates NUMBL, is also significantly downregulated in the LP group. NUMBL promotes lineage specification of mammary myoepithelial cells by inhibiting the Notch signaling pathway and the p53-p21 axis, influencing mammary duct elongation, side branch formation during puberty, epithelial cell maintenance, and lactation support [49]. In the ceRNA network, novel_circ_0000023, novel_circ_0000024, novel_circ_0000038, and novel_circ_0000040 all act as sponges, simultaneously sequestering bta-miR-193b and bta-miR-326. We speculate that these circRNAs promote target gene functions during lactation and contribute to mammary gland development. It is reported that circRNA-02191 promotes the generation of triglycerides and fatty acids in cow mammary gland cells by adsorbing miR-145 [50]. Circ 11103 enhanced the synthesis of triglyceride and fatty acid by adsorbing miR-128 in bovine gland cells [51]. CircRNA-08436 can increase the concentration of saturated fatty acids in goat mammary epithelial cells [52]. CircRNA-006258 can sequester miR-574-5p to regulate cell growth and milk synthesis in goat mammary epithelial cells [53]. Although the functions of circRNAs in mammary development and lactation have been reported in other species, their role in yaks remains unclear. We believe that through subsequent validation experiments, novel_circ_0000023, novel_circ_0000024, novel_circ_0000038, and novel_circ_0000040 will exhibit results similar to those observed in the aforementioned studies in yak mammary cells, providing valuable insights into the regulatory mechanisms of circRNAs involved in yak lactation and mammary gland development.

## 5. Conclusions

In this study, we analyzed the expression profiles of circRNAs in the mammary glands of lactating and dry-period yaks. We detected a total of 18,905 circRNAs. GO and KEGG analyses suggest that DECs may play roles in regulating milk synthesis and composition. CircRNAs in the ceRNA network may serve as promising targets for enhancing lactation and mammary gland development, with potential applications for improving milk production traits in yaks. Given the incomplete annotation of yak circRNA, this study offers valuable resources for enhancing the yak circRNA database and provides new insights into the regulatory mechanisms underlying lactation and mammary gland development in yaks.

## Figures and Tables

**Table 1:** Overview of RNA-Seq Data and Mapping Results.

Sample	Raw Reads	Clean Reads	Mapped Reads	Mapping Rate (%)	GC Content (%)	Q30 (%)
DP1	184,408,224	181,065,646	156,206,414	86.17	53.07	96.5
DP2	155,782,622	152,534,698	131,660,504	86.32	53.23	96.53
DP3	162,876,662	158,253,090	132,600,127	83.79	53.07	96.35
LP1	160,155,930	156,985,398	135,755,839	86.48	53.17	96.53
LP2	139,072,986	135,208,918	115,961,807	85.76	50.51	96.35

## Data Availability

circRNA raw sequencing data (SRA; PRJNA1186257) and miRNA raw sequencing data (SRA; PRJNA1186264) from our previous study were used in in this study. Due to the incomplete status of follow-up experiments, we will release the raw sequencing data of miRNAs one year from now. The detailed information was described in the main text.

## Supplementary Materials

The following supporting information can be downloaded at: https://www.mdpi.com/article/10.3390/ani15010089/s1, Figure S1: Pearson correlation between samples; Table S1: GO enrichment analyses of host genes for DECs; Table S2: KEGG enrichment analyses of host genes for DECs; Table S3: GO enrichment analyses of target genes of DEmiRNAs in the ceRNA network; Table S4: Primer sequences for transcripts used in real-time quantitative PCR.

## References

[1] Ding L., Wang Y., Kreuzer M., Guo X., Mi J., Gou Y., Shang Z., Zhang Y., Zhou J., Wang H. (2013). Seasonal Variations in the Fatty Acid Profile of Milk from Yaks Grazing on the Qinghai-Tibetan Plateau. J. Dairy Res..

[2] Guo X., Long R., Kreuzer M., Ding L., Shang Z., Zhang Y., Yang Y., Cui G. (2014). Importance of Functional Ingredients in Yak Milk-Derived Food on Health of Tibetan Nomads Living Under High-Altitude Stress: A Review. Crit. Rev. Food Sci. Nutr..

[3] Li H., Ma Y., Li Q., Wang J., Cheng J., Xue J., Shi J. (2011). The Chemical Composition and Nitrogen Distribution of Chinese Yak (Maiwa) Milk. Int. J. Mol. Sci..

[4] Richert M.M., Schwertfeger K.L., Ryder J.W., Anderson S.M. (2000). An Atlas of Mouse Mammary Gland Development. J. Mammary Gland. Biol. Neoplasia.

[5] Inman J.L., Robertson C., Mott J.D., Bissell M.J. (2015). Mammary Gland Development: Cell Fate Specification, Stem Cells and the Microenvironment. Development.

[6] Hennighausen L., Robinson G.W. (2005). Information Networks in the Mammary Gland. Nat. Rev. Mol. Cell Biol..

[7] Watson C.J., Khaled W.T. (2008). Mammary Development in the Embryo and Adult: A Journey of Morphogenesis and Commitment. Development.

[8] Sanger H.L., KLOTZt G., RIESNERt D., Gross H.J. (1976). Viroids Are Single-Stranded Covalently Closed Circular RNA Molecules Existing as Highly Base-Paired Rod-like Structures. Proc. Natl. Acad. Sci. USA.

[9] Kolakofsky D. (1976). Isolation and Characterization of Sendai Virus DI-RNAs. Cell.

[10] Rong D., Sun H., Li Z., Liu S., Dong C., Fu K., Tang W., Cao H. (2017). An Emerging Function of circRNA-miRNAs-mRNA Axis in Human Diseases. Oncotarget.

[11] Memczak S., Jens M., Elefsinioti A., Torti F., Krueger J., Rybak A., Maier L., Mackowiak S.D., Gregersen L.H., Munschauer M. (2013). Circular RNAs Are a Large Class of Animal RNAs with Regulatory Potency. Nature.

[12] Hansen T.B., Jensen T.I., Clausen B.H., Bramsen J.B., Finsen B., Damgaard C.K., Kjems J. (2013). Natural RNA Circles Function as Efficient microRNA Sponges. Nature.

[13] Wang D., Zhao Z., Shi Y., Luo J., Chen T., Xi Q., Zhang Y., Sun J. (2022). CircEZH2 Regulates Milk Fat Metabolism through miR-378b Sponge Activity. Animals.

[14] Liu Y., Hou J., Zhang M., Seleh-Zo E., Wang J., Cao B., An X. (2020). Circ-016910 Sponges miR-574-5p to Regulate Cell Physiology and Milk Synthesis via MAPK and PI3K/AKT–mTOR Pathways in GMECs. J. Cell. Physiol..

[15] Li X., Wu Y., Wang Y., Yang X., Gao R., Lu Q., Lv X., Chen Z. (2024). The Molecular Mechanism of circRNA-11228/miR-103/INSIG1 Pathway Regulating Milk Fat Synthesis in Bovine Mammary Epithelial Cells. Agriculture.

[16] Elnour I.E., Wang X., Zhansaya T., Akhatayeva Z., Khan R., Cheng J., Hung Y., Lan X., Lei C., Chen H. (2021). Circular RNA circMYL1 Inhibit Proliferation and Promote Differentiation of Myoblasts by Sponging miR-2400. Cells.

[17] Wang X., Cao X., Dong D., Shen X., Cheng J., Jiang R., Yang Z., Peng S., Huang Y., Lan X. (2019). Circular RNA TTN Acts As a miR-432 Sponge to Facilitate Proliferation and Differentiation of Myoblasts via the IGF2/PI3K/AKT Signaling Pathway. Mol. Ther. Nucleic Acids.

[18] Zhang X.-O., Wang H.-B., Zhang Y., Lu X., Chen L.-L., Yang L. (2014). Complementary Sequence-Mediated Exon Circularization. Cell.

[19] Martin M. (2011). Cutadapt Removes Adapter Sequences from High-Throughput Sequencing Reads. EMBnet.journal.

[20] Pearson W.R., Wood T., Zhang Z., Miller W. (1997). Comparison of DNA Sequences with Protein Sequences. Genomics.

[21] Kim D., Pertea G., Trapnell C., Pimentel H., Kelley R., Salzberg S.L. (2013). TopHat2: Accurate Alignment of Transcriptomes in the Presence of Insertions, Deletions and Gene Fusions. Genome Biol..

[22] Kim D., Salzberg S.L. (2011). TopHat-Fusion: An Algorithm for Discovery of Novel Fusion Transcripts. Genome Biol..

[23] Enright A.J., John B., Gaul U., Tuschl T., Sander C., Marks D.S. (2003). MicroRNA Targets in Drosophila. Genome Biol..

[24] Kruger J., Rehmsmeier M. (2006). RNAhybrid: microRNA Target Prediction Easy, Fast and Flexible. Nucleic Acids Res..

[25] Kertesz M., Iovino N., Unnerstall U., Gaul U., Segal E. (2007). The Role of Site Accessibility in microRNA Target Recognition. Nat. Genet..

[26] Wu X., Zhou X., Xiong L., Pei J., Yao X., Liang C., Bao P., Chu M., Guo X., Yan P. (2020). Transcriptome Analysis Reveals the Potential Role of Long Non-Coding RNAs in Mammary Gland of Yak During Lactation and Dry Period. Front. Cell Dev. Biol..

[27] Bu D., Luo H., Huo P., Wang Z., Zhang S., He Z., Wu Y., Zhao L., Liu J., Guo J. (2021). KOBAS-i: Intelligent Prioritization and Exploratory Visualization of Biological Functions for Gene Enrichment Analysis. Nucleic Acids Res..

[28] Kolberg L., Raudvere U., Kuzmin I., Adler P., Vilo J., Peterson H. (2023). G:Profiler—Interoperable Web Service for Functional Enrichment Analysis and Gene Identifier Mapping (2023 Update). Nucleic Acids Res..

[29] Untergasser A., Nijveen H., Rao X., Bisseling T., Geurts R., Leunissen J.A.M. (2007). Primer3Plus, an Enhanced Web Interface to Primer3. Nucleic Acids Res..

[30] Silver N., Best S., Jiang J., Thein S.L. (2006). Selection of Housekeeping Genes for Gene Expression Studies in Human Reticulocytes Using Real-Time PCR. BMC Mol. Biol..

[31] Zi X.-D., Luo B., Xia W., Zheng Y.-C., Xiong X.-R., Li J., Zhong J.-C., Zhu J.-J., Zhang Z.-F. (2018). Characterization of Transcriptional Complexity during Pre-Implantation Development of the Yak (Bos Grunniens) Using RNA-Seq. Reprod. Domest. Anim..

[32] Ma X., Guo X., Yongfu L., Wang T., Bao P., Chu M., Wu X., Yan P., Liang C. (2024). Identification of circRNA-Associated ceRNA Networks in the Longissimus Dorsi of Yak Under Different Feeding Systems. BMC Vet. Res..

[33] La Y., Ma X., Bao P., Chu M., Yan P., Liang C., Guo X. (2023). Genome-Wide Landscape of mRNAs, lncRNAs, and circRNAs during Testicular Development of Yak. Int. J. Mol. Sci..

[34] Liang Y., Gao Q., Wang H., Guo M., Arbab A.A.I., Nazar M., Li M., Yang Z., Karrow N.A., Mao Y. (2022). Identification and Characterization of Circular RNAs in Mammary Tissue from Holstein Cows at Early Lactation and Non-Lactation. Biomolecules.

[35] Wang J., Zhou H., Hickford J.G.H., Hao Z., Gong H., Hu J., Liu X., Li S., Shen J., Ke N. (2021). Identification and Characterization of Circular RNAs in Mammary Gland Tissue from Sheep at Peak Lactation and during the Nonlactating Period. J. Dairy Sci..

[36] Xuan R., Wang J., Li Q., Wang Y., Du S., Duan Q., Guo Y., He P., Ji Z., Chao T. (2023). Identification and Characterization of circRNAs in Non-Lactating Dairy Goat Mammary Glands Reveal Their Regulatory Role in Mammary Cell Involution and Remodeling. Biomolecules.

[37] Ma D., Zhao Y., Yu S., Zhang H., Cheng M., Cao H., Li Q., Min L. (2019). CircRNA as CeRNA Mediated by microRNA May Be Involved in Goat Lactation. Small Rumin. Res..

[38] Gonzalez-Iglesias A.E., Jiang Y., Tomić M., Kretschmannova K., Andric S.A., Zemkova H., Stojilkovic S.S. (2006). Dependence of Electrical Activity and Calcium Influx-Controlled Prolactin Release on Adenylyl Cyclase Signaling Pathway in Pituitary Lactotrophs. Mol. Endocrinol..

[39] Chiba T., Maeda T., Sanbe A., Kudo K. (2016). Serotonin Suppresses β-Casein Expression via PTP1B Activation in Human Mammary Epithelial Cells. Biochem. Biophys. Res. Commun..

[40] Chen Z., Zhou J., Wang M., Liu J., Zhang L., Loor J.J., Liang Y., Wu H., Yang Z. (2020). Circ09863 Regulates Unsaturated Fatty Acid Metabolism by Adsorbing miR-27a-3p in Bovine Mammary Epithelial Cells. J. Agric. Food Chem..

[41] Chen Z., Cao X., Lu Q., Zhou J., Wang Y., Wu Y., Mao Y., Xu H., Yang Z. (2021). Circ01592 Regulates Unsaturated Fatty Acid Metabolism through Adsorbing miR-218 in Bovine Mammary Epithelial Cells. Food Funct..

[42] Xu B., Wang M., Zhang X., Ha S., Wang C., Ao C., Wang H. (2012). Arginine levels affect growth and CSN3 gene expression of dairy cows mammary epithelial cells in vitro. Chin. J. Anim. Nutr..

[43] Wang M., Xu B., Wang H., Bu D., Wang J., Loor J.-J. (2014). Effects of Arginine Concentration on the In Vitro Expression of Casein and mTOR Pathway Related Genes in Mammary Epithelial Cells from Dairy Cattle. PLoS ONE.

[44] Wu G., Bazer F.W., Hu J., Johnson G.A., Spencer T.E. (2005). Polyamine Synthesis from Proline in the Developing Porcine Placenta1. Biol. Reprod..

[45] Da Rosa F.T., Moreira C.G.A., Barbero M.M.D., Hurtado Lugo N.A., de Camargo G.M.F., Aspicueta Borquis R.R., de Oliveira H.N., Boligon A.A., de Vargas L., Moreira H.L.M. (2020). Associations between MUC1 Gene Polymorphism and Resistance to Mastitis, Milk Production and Fertility Traits in Murrah Water Buffaloes. J. Appl. Anim. Res..

[46] Yonezawa T., Haga S., Kobayashi Y., Katoh K., Obara Y. (2009). Short-Chain Fatty Acid Signaling Pathways in Bovine Mammary Epithelial Cells. Regul. Pept..

[47] Gerlando R.D., Sutera A.M., Mastrangelo S., Tolone M., Portolano B., Sottile G., Bagnato A., Strillacci M.G., Sardina M.T. (2019). Genome-Wide Association Study between CNVs and Milk Production Traits in Valle Del Belice Sheep. PLoS ONE.

[48] Dai W., Zou Y., White R.R., Liu J., Liu H. (2018). Transcriptomic Profiles of the Bovine Mammary Gland during Lactation and the Dry Period. Funct. Integr. Genom..

[49] Zhang Y., Li F., Song Y., Sheng X., Ren F., Xiong K., Chen L., Zhang H., Liu D., Lengner C.J. (2016). *Numb* and *Numbl* Act to Determine Mammary Myoepithelial Cell Fate, Maintain Epithelial Identity, and Support Lactogenesis. FASEB J..

[50] Chen Z., Wang Y., Wang K., Zhang Z., Han M., Li G., Zhang B., Yang Y., Loor J.J., Yang Z. (2023). CircRNA-02191 Regulating Unsaturated Fatty Acid Synthesis by Adsorbing miR-145 to Enhance CD36 Expression in Bovine Mammary Gland. Int. J. Biol. Macromol..

[51] Chen Z., Lu Q., Liang Y., Cui X., Wang X., Mao Y., Yang Z. (2021). Circ11103 Interacts with miR-128/PPARGC1A to Regulate Milk Fat Metabolism in Dairy Cows. J. Agric. Food Chem..

[52] Wang Y., Wu Y., Yang S., Gao R., Lv X., Yang Z., Jiao P., Zhang N., Loor J.J., Chen Z. (2024). m6A Methylation Mediates the Function of the circRNA-08436/miR-195/ELOVL6 Axis in Regards to Lipid Metabolism in Dairy Goat Mammary Glands. Animals.

[53] Zhang M., Ma L., Liu Y., He Y., Li G., An X., Cao B. (2020). CircRNA-006258 Sponge-Adsorbs miR-574-5p to Regulate Cell Growth and Milk Synthesis via EVI5L in Goat Mammary Epithelial Cells. Genes.

