# Supplementary figures and images for: Identification and Analysis of Circular RNAs in Mammary Gland from Yaks Between Lactation and Dry Period
Source: Animals (Basel). 2025 Jan 3;15(1):89. doi: 10.3390/ani15010089 (PMC11718809; doi:10.3390/ani15010089)

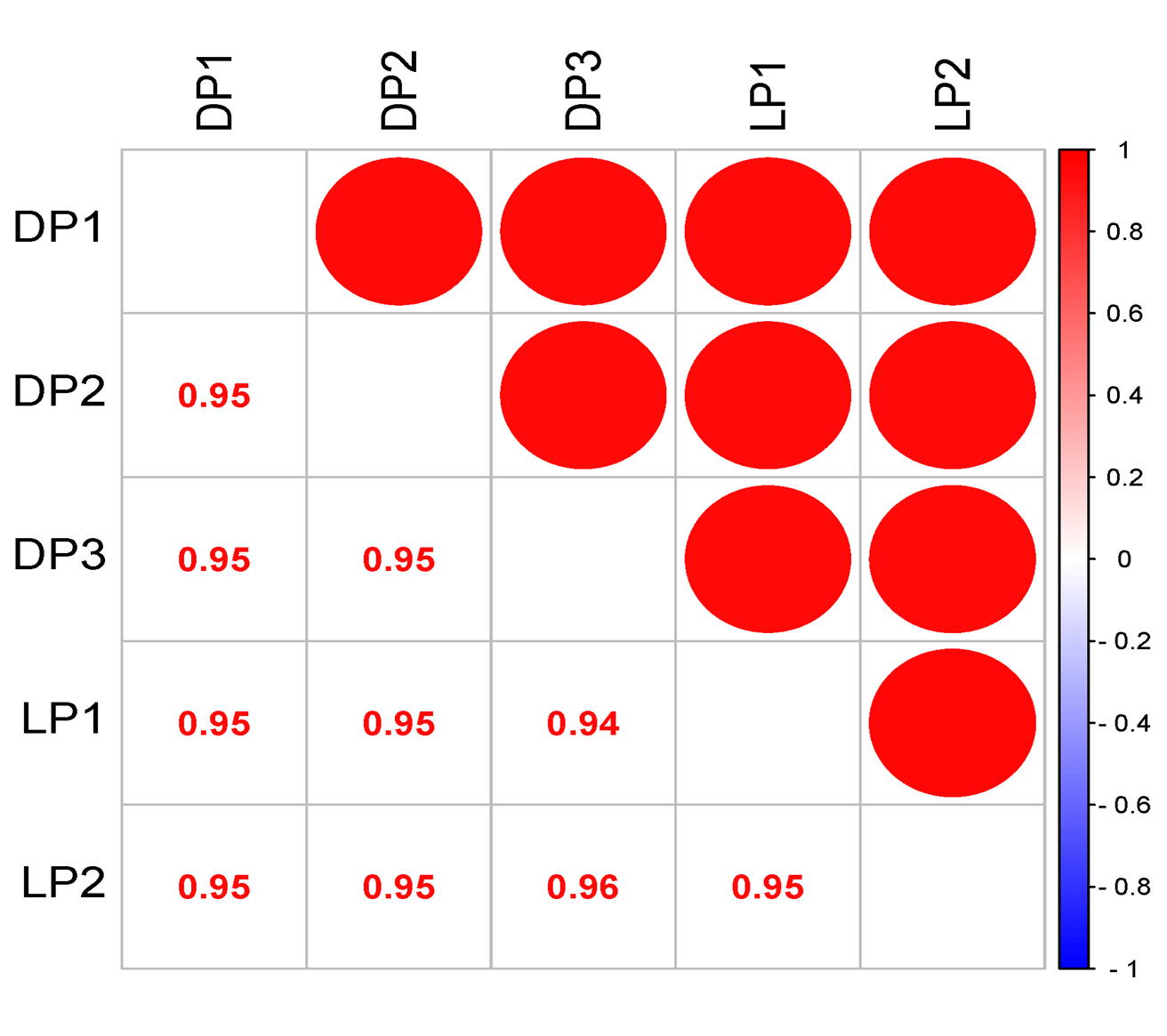

Supplement: Supplementary file 1 [file animals-15-00089-s001.zip › supplementary file/Figure S1. Pearson correlation between samples.png]
